# Prenatal diagnosis and treatment of fetal intestinal volvulus: A case report

**DOI:** 10.1097/MD.0000000000041337

**Published:** 2025-01-17

**Authors:** Xiaoyan Zeng, Xiang Li, Weinong Mo, Xuefen Zhu

**Affiliations:** a Department of Neonatology, Hangzhou Women’s Hospital, Hangzhou, Zhejiang, China; b Department of Pediatric Surgery, Hangzhou Children’s Hospital, Hangzhou, Zhejiang, China.

**Keywords:** fetal intestinal volvulus, magnetic resonance imaging, prenatal diagnosis, ultrasound, Whirlpool sign

## Abstract

**Rationale:**

Fetal intestinal volvulus, a rare and severe disorder, poses significant diagnostic challenges prenatally and can lead to intrauterine death or adverse neonatal outcomes if untreated in a timely manner. This study reports a case of fetal intestinal volvulus confirmed postoperatively, providing insights into its clinical manifestations, diagnostic methods, and treatment outcomes, thereby enhancing understanding of this rare condition.

**Patient concerns:**

A Chinese gravida 2, para 1 female presented at 32 weeks and 5 days of gestation with decreased fetal movements. Fetal ultrasound and MRI revealed small intestinal torsion and obstruction. Due to fetal distress, an emergency cesarean section was performed, followed by an urgent laparotomy on the neonate to untwist the intestinal volvulus and resect the necrotic bowel segment.

**Diagnosis:**

Fetal intestinal volvulus

**Interventions:**

Following an emergency cesarean section, the neonate was promptly transferred to the surgical department for immediate surgical intervention. The procedure encompassed detorsion of the intestinal volvulus and resection of the necrotic bowel segment.

**Outcomes:**

The infant exhibited a satisfactory postoperative recovery, successfully transitioned to complete oral and gastrointestinal nutrition, and demonstrated normal physical growth and cognitive development during the ensuing follow-up period.

**Lessons:**

Fetal intestinal volvulus requires multidisciplinary collaboration for diagnosis and treatment. Early recognition, prompt delivery, and urgent surgical intervention are crucial for optimizing neonatal outcomes. By sharing this case, we hope to enhance the diagnostic and therapeutic capabilities of healthcare providers in managing this rare but severe condition.

## 1. Introduction

Fetal intestinal volvulus, a rare and potentially fatal surgical condition, currently lacks a precise incidence rate.^[1]^ Its prenatal diagnosis remains challenging due to the absence of distinct clinical manifestations and specific ultrasonographic markers. Delayed intervention can result in extensive intestinal necrosis and potentially fetal demise.^[2,3]^

In this case report, we present a fetus with prenatal ultrasonographic and MRI findings of intestinal dilation and the Whirlpool sign. Intraoperative confirmation revealed ileal torsion. By sharing this case, we aim to enhance clinicians’ diagnostic and therapeutic capabilities in managing fetal intestinal volvulus.

## 2. Case presentation

A 32-year-old Chinese gravida 2, para 1 woman presented to our hospital at 32 weeks and 5 days of gestation due to a 6-hour decrease in fetal movements. Previous antenatal exams were unremarkable, with the last one conducted at 32 weeks. Upon admission, emergency fetal ultrasound revealed no apparent abnormalities. However, concerns arose due to suspicious findings on cardiotocography, prompting a repeat fetal ultrasound at 22 hours. This revealed dilated intestinal segments, local tortuosity with a Whirlpool sign, and a small amount of abdominal fluid. Concurrent MRI scanning demonstrated significant dilation of the small intestine in the mid-abdomen, with a maximum dilated bowel diameter of approximately 13.3 mm. The MRI also showed a Whirlpool sign and a minor liquid signal shadow in the abdominal cavity. The diagnosis was small intestinal obstruction, intestinal torsion, and abdominal fluid accumulation.

After a multidisciplinary consultation involving obstetrics, NICU, and pediatric surgery, and in consultation with the patient, she elected to continue with labor. Premature rupture of membranes occurred 34 hours after admission, and cardiotocography demonstrated an unresponsive pattern. An emergency cesarean section was performed, delivering a male neonate with clear amniotic fluid (volume < 50 mL) and a birth weight of 2080 g. The neonate exhibited an Apgar score of 10 at both 1 and 5 minutes. Upon examination, the abdomen was notably distended, and bowel sounds were absent. After intubation and ventilator-assisted ventilation, the neonate was transferred to a children’s hospital for further treatment.

**Figure 1.**
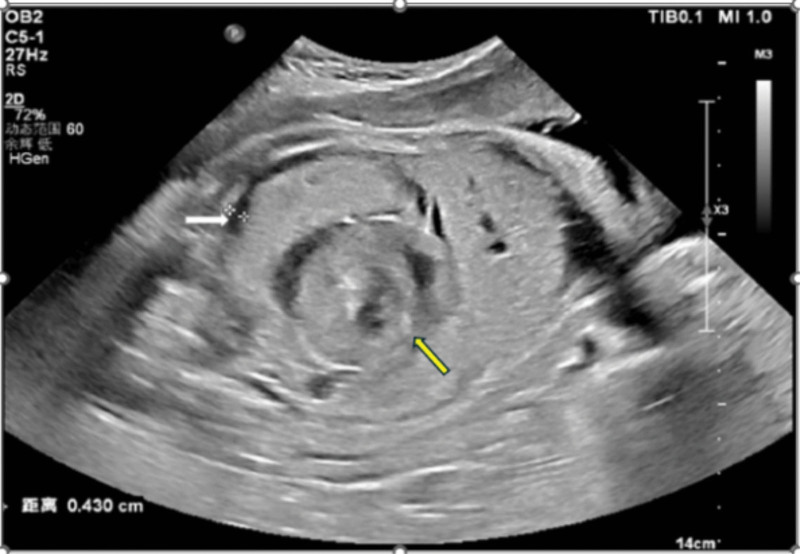
Fetal ultrasound revealed intestinal dilation with ascites (white arrow), Whirlpool sign (yellow arrow).

**Figure 2.**
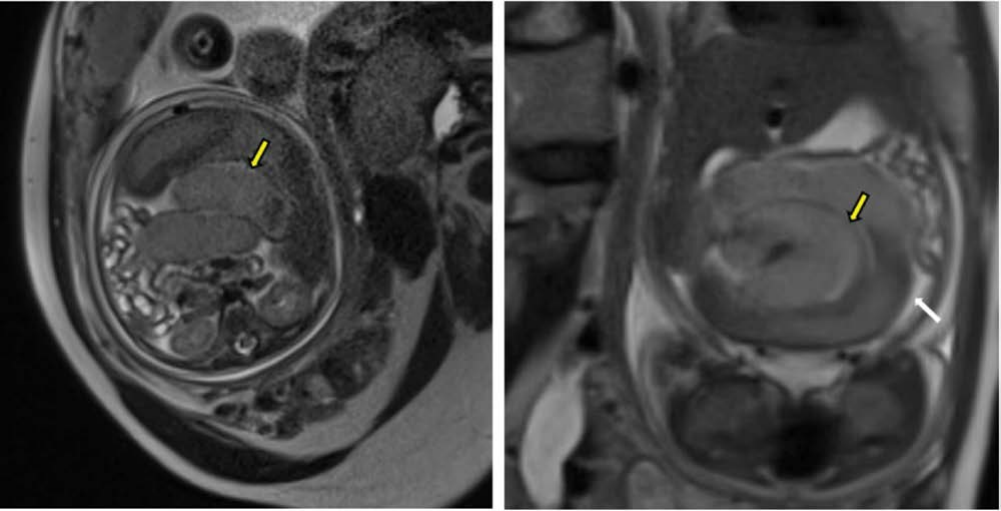
The fetal MRI Showed that the intestinal was spirally dilated (yellow arrow), and the ascites was visible (white arrow). MRI = magnetic resonance imaging.

**Figure 3.**
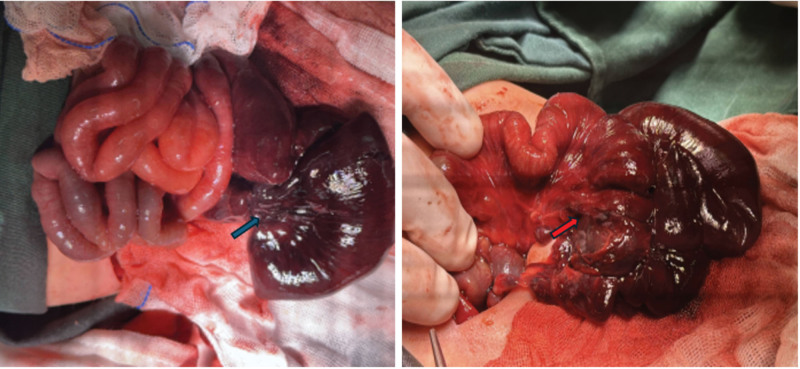
Ileum torsion (blue arrow), bowel necrosis, mesenteric defect (red arrow).

An exploratory laparotomy was performed 11 hours after birth, revealing rotation of the intestinal loops by 2 complete turns from the mesenteric root. Portions of the bowel exhibited ischemia and bloody ascites. Surgical correction of the volvulus was performed. Approximately 40 cm of necrotic intestine was resected, and a terminal ileostomy was created. The proximal end of the necrotic bowel lay at approximately 85 to 90 cm from the Treitz ligament, while the distal end was situated 2.0 cm from the ileocecal valve. Postoperatively, the patient was managed jointly by the NICU and pediatric surgery teams. On postoperative day 8, the patient was initiated on enteral nutrition utilizing a lactose-free, deeply hydrolyzed formula. The volume of the formula was gradually increased, and by postoperative day 19, the patient had successfully transitioned to full enteral nutrition. On postoperative day 45, the patient was discharged with a drainage tube in situ. At discharge, the patient’s weight was 2.9 kg, and specific instructions were provided for oral administration of 45 mL of the formula every 2 hours, with a daily drainage output of 110 to 150 mL. Three months postoperation, the patient’s weight increased to 4.4 kg, and the drainage tube was subsequently removed. Currently, at 8 months of age, the patient’s physical growth and mental development are normal (Figs. 1–3).

## 3. Discussion

Fetal intestinal volvulus is a spiral rotation of the intestine around the mesentery and mesenteric vessels, which leads to compromised venous return and intestinal expansion. In severe cases, intestinal perforation, peritonitis, and even intrauterine fetal death can occur due to ischemic necrosis of the intestinal wall.^[3,4]^ Fetal intestinal volvulus generally occurs in the middle to late stages of pregnancy, with a peak incidence after 30 weeks of gestation. Montironi et al^[1]^ reported that prenatal suspicion or diagnosis by ultrasound typically occurs between 15 and 39 weeks of gestation, averaging at 30 weeks. Bartholmot et al^[5]^ further identified 2 peaks in the diagnosis of fetal volvulus, occurring at approximately 27 and 32 weeks of gestation respectively. The presented case was diagnosed with small intestinal volvulus at 32 weeks and 5 days of gestation.

Currently, the etiology of fetal intestinal volvulus remains unclear. The majority of cases are idiopathic, while others may be secondary to conditions such as intestinal malrotation, fibrocytes, gastrointestinal defects, intestinal atresia, immature development of ganglion cells in the colon wall, and mesenteric defects.^[1,6]^ In the presented case, mesenteric torsion was observed at approximately 720°, along with a mesenteric defect. We consider that the etiology of this case is idiopathic, and the mesenteric defect was a consequence of mesenteric ischemic necrosis subsequent to torsion, rather than being the primary cause of intestinal torsion.

Currently reported cases of fetal intestinal volvulus were mostly discovered through ultrasonography during routine prenatal checkups, decreased fetal movements, or abnormal fetal heart monitoring.^[1]^ As a noninvasive, cost-effective, and timely prenatal screening tool, ultrasound can be repeated multiple times. Ultrasonic imaging of intestinal dilatation, the whirlpool sign, and the coffee bean sign exhibit high specificity and sensitivity in diagnosing fetal intestinal volvulus.^[7]^ When typical ultrasonographic images are lacking, MRI can serve as an effective complementary method for prenatal diagnosis of fetal intestinal volvulus due to its high soft tissue resolution, which aids in identifying abnormal tissue attributes.^[8]^ In this present case, initial ultrasound examination of the fetus revealed no abnormalities, but a follow-up ultrasound the next day demonstrated a “whirlpool-like” alteration in the fetal bowel loops. MRI was subsequently performed to aid in diagnosis. The accuracy of ultrasound in detecting fetal intestinal volvulus depends not only on the typicality of intestinal manifestations but also on the examiner’s proficiency and knowledge of this disease. Therefore, it is crucial to raise awareness among ultrasound physicians regarding fetal intestinal volvulus and establish ultrasound diagnostic criteria for this condition. MRI, with its advantages of high resolution and multi-planar imaging, provides more 3-dimensional and clear images, thereby enhancing diagnostic accuracy. The authors recommend employing MRI when feasible to improve the diagnostic accuracy of fetal intestinal volvulus.

The prognosis of fetal intestinal volvulus depends on multiple factors, including gestational age, birth weight, the extent of necrotic bowel, and associated abnormalities.^[9]^ Therefore, accurate diagnosis of fetal intestinal volvulus, selection of appropriate delivery timing, and timely surgical intervention are all crucial.^[10]^ Continuous ultrasound monitoring is necessary to assess bowel condition and determine whether termination of pregnancy is warranted. Currently, however, there is no consensus on the optimal delivery timing for fetal intestinal volvulus.^[2,5]^ Some viewpoints suggest that fetuses with a gestational age exceeding 34 weeks should undergo immediate delivery upon ultrasound evidence of bowel deterioration. For fetuses younger than 34 weeks with normal fetal movement and fetal heart monitoring, pregnancy can be continued with intensified monitoring.^[1,5]^ In this case, after diagnosing intestinal volvulus via ultrasound and MRI, the family opted to continue the pregnancy until fetal distress was detected through fetal heart monitoring, followed by a cesarean section. A Multidisciplinary Team involving obstetrics, neonatology, and neonatal surgery was assembled before delivery. The neonate underwent surgical treatment 11 hours postdelivery. Although the child survived, 40cm of necrotic bowel had to be resected. Had the newborn been delivered prior to the onset of fetal distress, a greater length of bowel could have potentially been preserved, thereby enhancing the newborn’s quality of life. Hence, further deliberation is warranted on determining the optimal delivery timing in cases of fetal intestinal volvulus.

This case report details the diagnosis and treatment of fetal intestinal volvulus in 1 patient, with inherent limitations in generalizability. Diagnostic challenges arose from the condition’s rarity and prenatal imaging limitations, and institutional variations in equipment, techniques, and experience may further affect case management.

## 4. Conclusion

The diagnosis and treatment of fetal intestinal volvulus demands multidisciplinary collaboration. Enhancing ultrasonographers’ comprehension of this disease is beneficial for early detection. If conditions allow, fetal MRI can aid in the diagnostic process. Obstetricians must carefully assess gestational age, fetal status, and ultrasound findings to ascertain the most suitable time for delivery. After delivery, the comprehensive care by the NICU, pediatric surgery, and anesthesiology teams, coupled with prompt surgical intervention, can significantly reduce fetal mortality and enhance newborns’ quality of life.

## Author contributions

**Data curation:** Weinong Mo, Xuefen Zhu.

**Formal analysis:** Xiaoyan Zeng, Xiang Li, Xuefen Zhu.

**Funding acquisition:** Xiaoyan Zeng, Xiang Li, Weinong Mo.

**Investigation:** Weinong Mo.

**Resources:** Xiang Li.

**Writing – original draft:** Xiaoyan Zeng, Xuefen Zhu.

**Writing – review & editing:** Xiaoyan Zeng, Xuefen Zhu.
